# Supplementary material for: Activation of Neutrophils by Mucin–Vaterite Microparticles
Source: Int J Mol Sci. 2022 Sep 13;23(18):10579. doi: 10.3390/ijms231810579 (PMC9501559; doi:10.3390/ijms231810579)
Supplement: Supplementary file 1 [file ijms-23-10579-s001.zip › ijms-1886865-supplementary.pdf]

Supplementary data

ACTIVATION OF NEUTROPHILS BY MUCIN-VATERITE MICROPARTICLES

Mikhalchik E.V. [1], Basyreva L.Yu. [1], Gusev S.A. [1], Panasenko O.M. [1], Klinov D.V. [1,5], Barinov N.A.[1,5], Morozova O.V. [1,3], Moscalets A.P.[1,5], Maltseva L.N. [2], Filatova L.Yu. [2], Pronkin E.A. [1], Bespyatykh J.A. [1,4], Balabushevich N.G. [2]

[1] Federal Research and Clinical Center of Physical-Chemical Medicine of Federal Medical Biological Agency, Moscow, Russia

[2] Lomonosov Moscow State University, Faculty of Chemistry, Moscow, Russia

[3] National Research Center of Epidemiology and Microbiology of N.F. Gamaleya, Moscow, Russia

[4]Mendeleev University of Chemical Technology of Russia, Moscow, Russia

[5]Sirius University of Science and Technology, Sochi, Russia

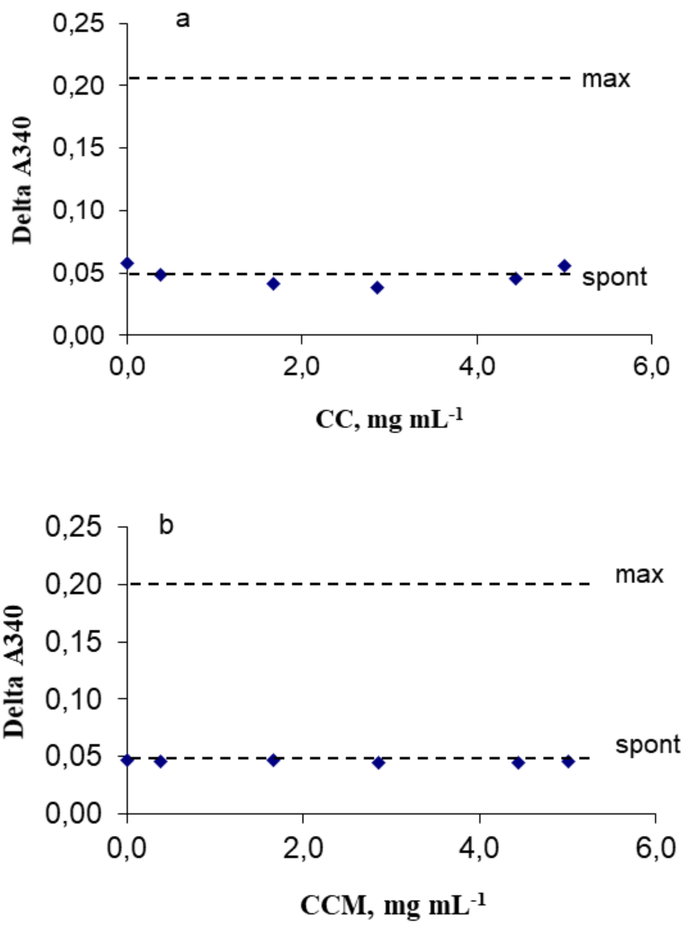

Figure S1. LDH activity in extracellular medium after neutrophils incubation with microparticles for 30 min at 37°C.

Cells and particles were sedimented by centrifugation for 10 min at 900 g and LDG activity was assayed using LDH-Olvex assay kit (Olvex, St-Petersburg, Russia). LDH activity was evaluated by decrease in $NADPH_2$ optical absorbance detected at 340 nm. Maximal value was assayed after total lysis of the cells using freezing-thawing procedure in water. Spontaneous LDH activity values were registered in cell suspension without particles (0.15 M NaCl was added instead).

As data in Fig. show, no lysis was detected after incubation of neutrophils with 5 mg mL^{-1} CC or CCM at 37°C for 30 min.

However, we could not exclude effects of direct adsorption of LDH onto the particles which should significantly influence the results. So, we also assessed functional activity of neutrophils within the time of CL-registration. Neutrophils were activated with CC or CCM in various concentrations, and after peak value of Luc-CL was reached, phorbol 12-myristate 13-acetate (0.156 μM) was added, and the second peak corresponded to PMA-induced superoxide generation (Fig.S2).

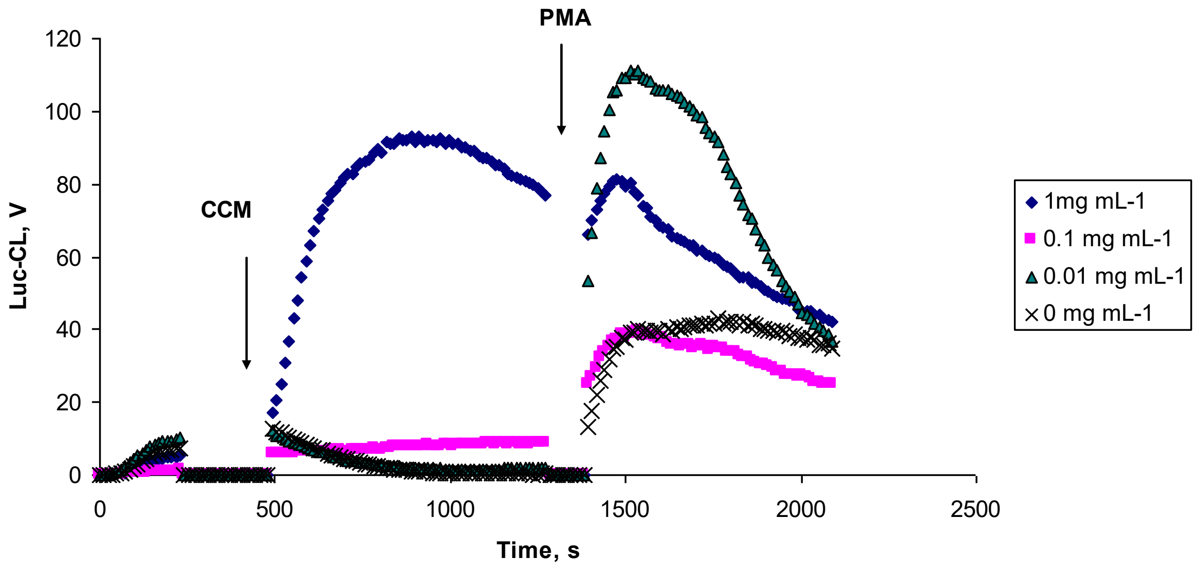

Figure S2. Typical time-courses of Luc-CL response of neutrophils stimulated with various CCM-concentrations followed by PMA addition. Concentration or neutrophils was 0.5 mln mL^{-1}.

Then light sum of Luc-CL response was calculated as an area under each curve, starting from the moment of particles addition. The results both for CC and CCM are represented in Fig. S3.

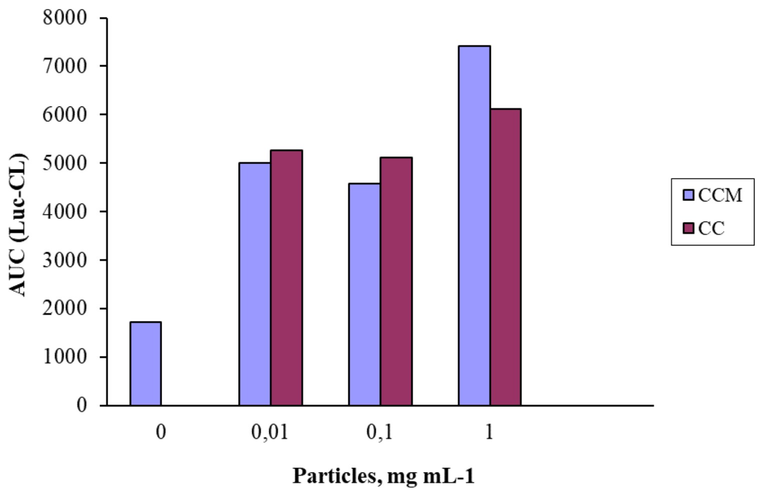

Figure S3. Light sums of Luc-CL responses of neutrophils stimulated with various concentrations of CC or CCM followed by PMA. The values were calculated as area under each time-course curve (AUC), starting from the moment of particles addition (Fig S2).

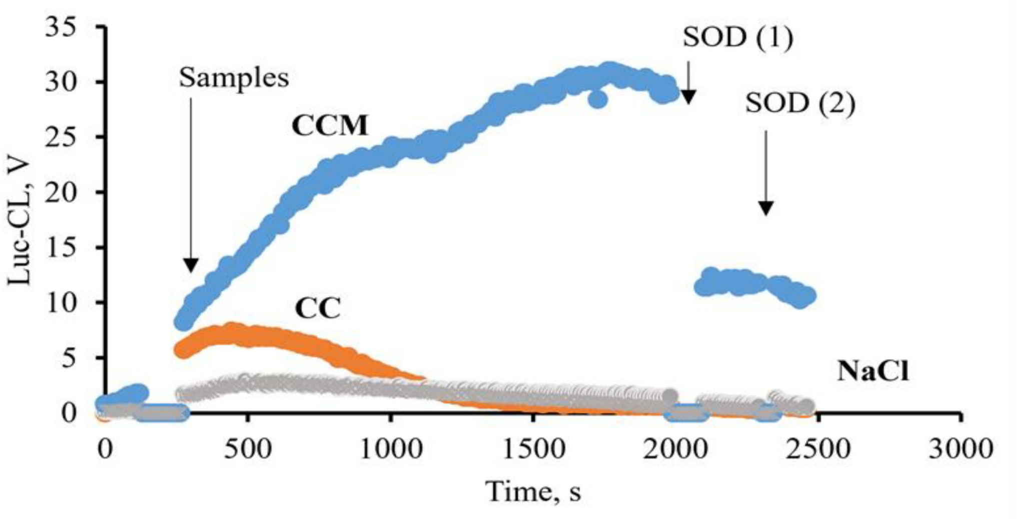

Figure S4. Typical time-course of neutrophil Luc-CL response stimulated with CC or CCM. SOD was added in two divided doses up to 15 U mL-1 (1) and 30 U mL-1 (2).

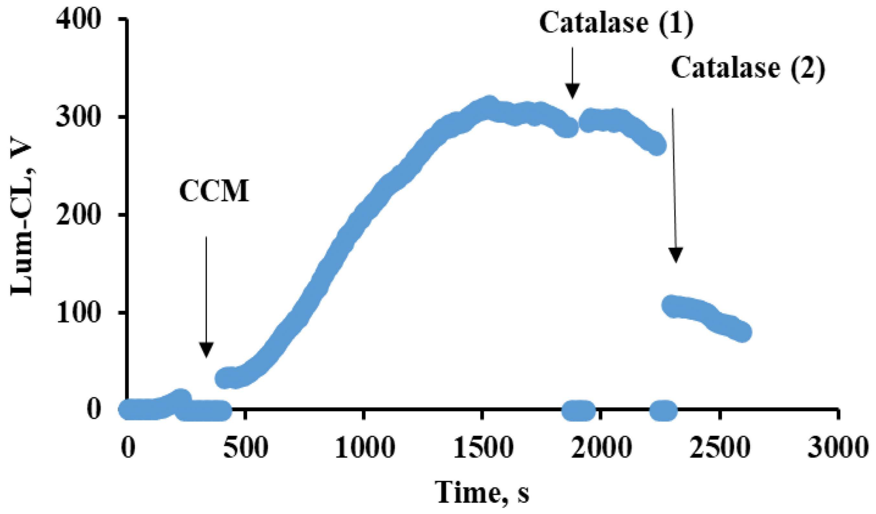

Figure S5. Typical time-course of neutrophil Lum-CL response stimulated with CCM. Catalase was added in two divided doses up to 500 U mL-1 (1) and 10000 U mL-1 (2).

According to RT^2-PCR data, incubation with CC or CCM for 1 h did not influence expression of IL-6, IL-8 or IL-10 genes in neutrophils (Table S1).

Table S1. Cytokine gene expression in neutrophils incubated with CC, CCM and NaCl (delta Ct).

Activator	IL-6	IL-8	IL-10
NaCl	16.5	21.8	23.4
CC	16.8	23.6	22.1
CCM	17.0	21.2	23.7

No significant difference between control neutrophils (NaCl) and samples with CC or CCM was found as the change were within 10%.

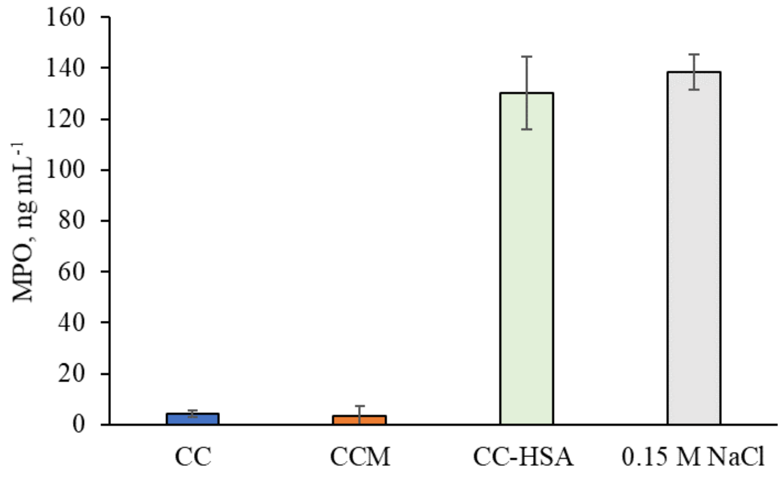

Figure S6. Concentration of MPO after incubation of its solution (120 ng mL^{-1}) with 5 ng mL^{-1} CC, CCM, CC-HAS* microparticles or 0.15 M NaCl for 1 h at 37°C.

*5 mg mL^{-1} CC microparticles were treated with 5 mg mL^{-1} human serum albumin (HSA) (under the same conditions as with mucin including washing with 0.15 M NaCl)

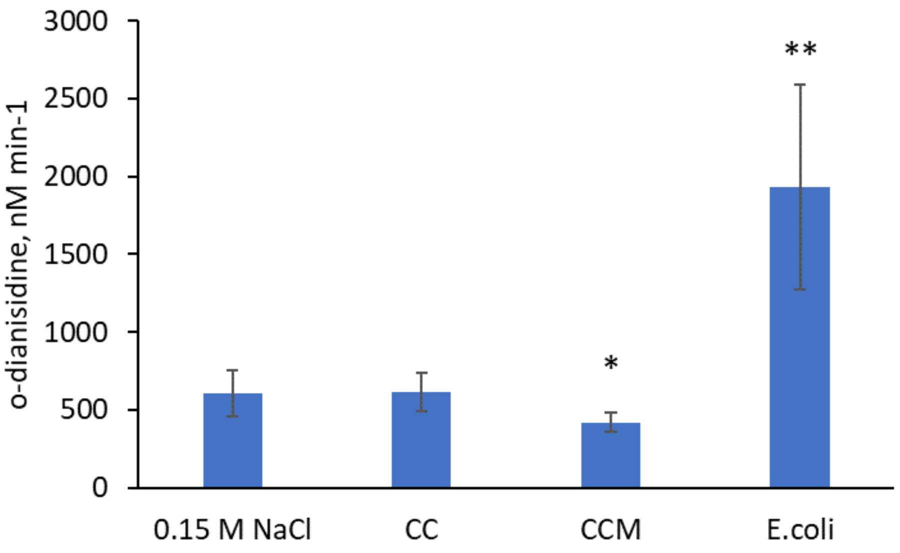

Figure S7. MPO activity in extracellular medium after incubation of neutrophils with the samples, measured using o-dianisidine. *$p < 0.05$ vs CC and 0.15 M NaCl. ** $p < 0.05$ vs CC, CCM or 0.15 M NaCl

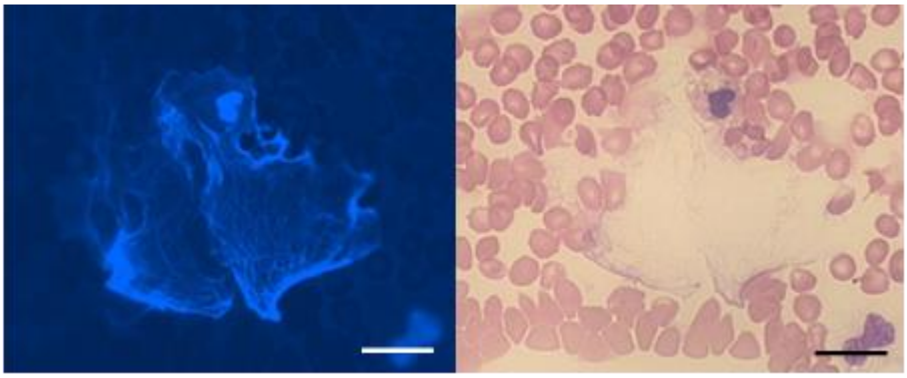

(a) (b)

Figure S8. Neutrophil extracellular trap: (a) stained with Hoechst dye, fluorescent microscopy; (b) stained with Romanowsky dye, light microscopy. Scale bar is 30 µm.

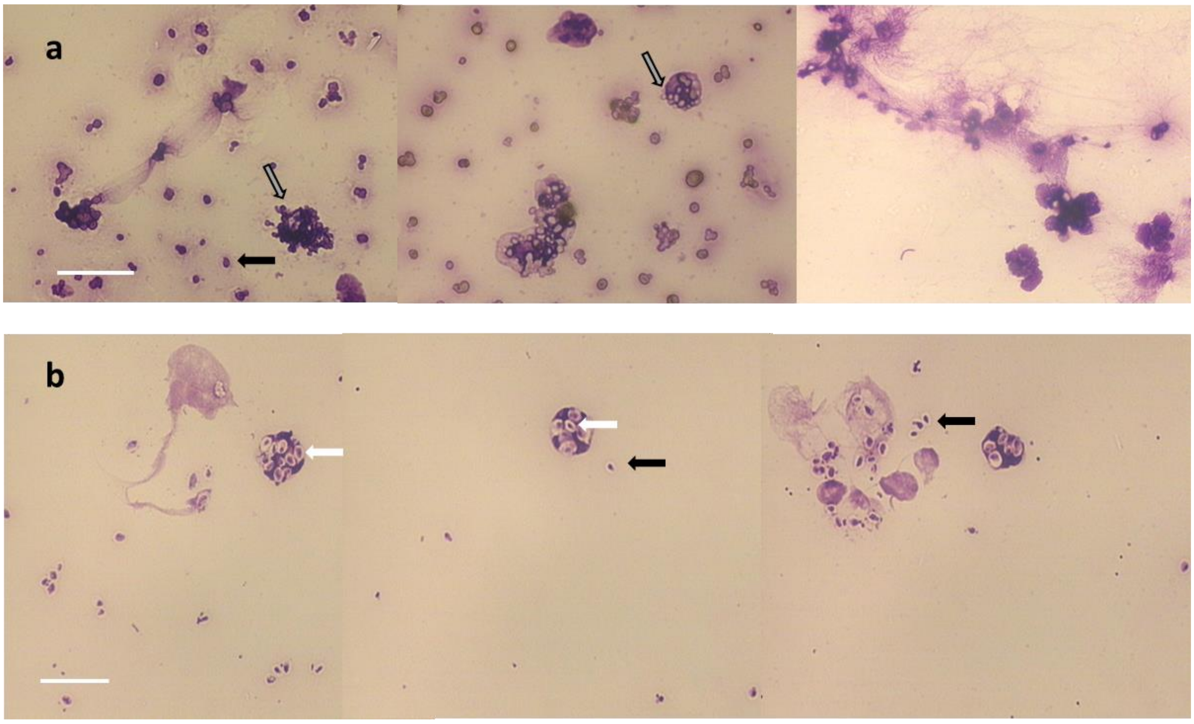

Figure S9. Microphotographs of smears stained with Romanowsky dye: (a) neutrophils incubated with CCM; (b) neutrophils incubated with zymosan. Scale bar is 30 µm. White arrows indicate phagocytosed particles, grey arrows – adhered particles, black arrows – free particles.

Zymosan can be well seen within phagolysosomes inside neutrophils, while CCM microparticles are rather bound to neutrophil surface. Neutrophil aggregation and formation of NET-like structures is well seen in Fig. S9 a.
